# Are some controversial views in bioethics Juvenalian satire without irony?

**DOI:** 10.1007/s11017-022-09604-0

**Published:** 2022-12-24

**Authors:** Matti Häyry

**Affiliations:** Aalto University School of Business, Espoo, Finland

**Keywords:** Controversy, Utilitarian, Dogmatic, Juvenalian, Satire, Irony, Humor

## Abstract

The article examines five controversial views, expressed in Jonathan Swift’s *A Modest Proposal*, Helga Kuhse and Peter Singer’s *Should the Baby Live? The Problem of Handicapped Infants*, Alberto Giubilini and Francesca Minerva’s “After-birth abortion: why should the baby live?”, Julian Savulescu’s “Procreative beneficence: why we should select the best children”, and the author’s “A rational cure for prereproductive stress syndrome”. These views have similarities and differences on five levels: the grievances they raise, the proposals they make, the justifications they explicitly use, the justifications they implicitly rely on, and the criticisms that they have encountered. A comparison of these similarities and differences produces two findings. First, some controversial views based on utilitarian considerations would probably fare better flipped upside down and presented as Juvenalian satires. Secondly, a modicum of humor or modesty could help presenters of controversial views to stir polite critical discussion on the themes that they put forward.

## Introduction

The following five theses have similarities and differences:

A. Poor people should be allowed to sell their babies as food for rich people, because this would alleviate the problem of poverty.
B. Severely disabled babies should be painlessly killed because they are a burden to others and human existence with only suffering has no value.
C. Mothers should be allowed to kill their babies by proxy if they feel that they can neither live with their offspring nor give them up for adoption.
D. Genetically imperfect babies should not exist, and potential parents should be persuaded that they should always have the best children they can.
E. Babies should not exist, and potential parents should be persuaded that human reproduction can lead to the creation of miserable lives.

The theses are similar in that they all involve babies. They are also similar in that they are controversial, or at least prone to meet opposition at some level. They are different in many senses. The first, A, is from a historical satire, whereas the others have been presented in bioethical discussion during the last decades. The second, B, is almost accepted in clinical practice, albeit in an indirect and implicit form. The third, C, has reportedly engendered more threats towards its authors than the others, although B competes in the same category. The fourth and fifth, D and E, do not involve killing babies like the other three. They differ from each other in that D is eugenic (against the creation of allegedly imperfect offspring), while E is antinatalist (against all human reproduction).

In what follows, I will begin by describing the theses and their implications. I will then compare their salient features and try to gauge in which sense they are controversial. After a few remarks on utilitarianism, I will suggest that deliberate controversy may in some cases be seen as Juvenalian satire without irony. Since irony is the redeeming element of satire, my conclusion will be that distancing and humor might have a place in the presentation of controversial views.

## A modest proposal: eating children

Jonathan Swift published in 1729, anonymously, his satirical pamphlet *A Modest Proposal for Preventing the Children of Poor People from Being a Burthen to their Parents, or the Country, and for Making them Beneficial to the Publick*, better known just as *A Modest Proposal* [1]. The proposer, to be distinguished from Swift, describes the miserable conditions in which poor people live in Ireland, observes how starving Catholic families cannot properly care for their children, and then suggests that the issue could be solved by fattening the children up, slaughtering them, and selling them as food to the rich. The proposer goes on to cite statistical evidence for the economic feasibility of the suggestion and to provide recipes for new dishes.

Swift’s political agenda in the pamphlet was to draw attention to the plight of the Irish poor; the indifference of affluent people in the face of this plight; and the exploitation of the Irish by the English. An underlying theoretical point is that detached calculations cannot legitimate measures that involve clearly perceived immorality. As a criticism of prevailing economic ideas, the pamphlet goes against the mercantilist belief that people are the riches of a nation and against the Enlightenment conviction that all problems can be solved by innovative plans for social engineering. The adjacent moral criticism is that an unquestioned faith in economic solutions commodifies poor people and makes them mere instruments for the flourishing of the elite [2–4].

We have here, then, the following elements. (1) An explicit depiction of a horrid social situation. (2) A mock suggestion for improving the situation by cannibalism and using the children of the Irish poor as a mere means to an economic and culinary end. (3) A mock justification for the suggestion in terms of the wellbeing of the poor and the wealth of the nation. (4) Underneath, Swift’s implicit moral condemnation of such calculating, pseudo-objective endeavors to deal with social issues. Comparisons with the other four theses, B-E, will show how this satirical treatment of a topic differs from current presentations of controversial ideas.

## A modern classic: killing babies with severe disabilities

Helga Kuhse and Peter Singer stirred the medical ethics community in 1985 with their book *Should the Baby Live? The Problem of Handicapped Infants* [5]. They started by arguing that severely disabled children are a burden to their parents and to society. The parents’ lives are overshadowed by their caring duties, they will not in the majority of cases dare have more children, and care in institutions can be so expensive that at the cost of one severely disabled inpatient in an affluent country numerous disadvantaged children could be helped to live decent lives in developing nations. In addition to the burden to others, the children’s existence can be so miserable that even medical professionals sometimes decide to let them die soon after birth. Kuhse and Singer argued that letting these infants die should be further encouraged, and that if waiting for nature to take its course prolongs futile suffering, they should be actively and mercifully killed. Despite the illegality, the latter practice was not unknown to pediatricians, although an air of secrecy and paternalism surrounded it [6, 7]. Kuhse and Singer attributed the secrecy and hesitation to the doctrine of sanctity of life.

The authors went on to criticize that doctrine, partly due to its speciesism. If societies accept the slaughter of pigs, cattle, and chickens on mass scale, they are not seriously committed to protecting all life, only the lives of human beings. Kuhse and Singer did not see, however, how species membership could be the decisive factor in matters of life and death. After rejecting attempts to prioritize humanity, they concluded that letting infants with disabilities die or killing them cannot be neutrally prohibited by appeals to the sanctity-of-life doctrine.

The elements of this case bear similarities with Swift’s proposal, but there are also notable differences. (1) The description of the emotional and economic burden and the suffering of some babies was real enough to make the matter worthy of consideration. This makes the case, to a certain extent, comparable to Swift’s plea for the plight of the Irish poor. (2) The recommendation to end the lives of some infants with disabilities was genuine, and Kuhse and Singer gave a detailed account of the criteria to be used. The careful explication of the case makes them akin to Swift’s proposer, but unlike Swift, Kuhse and Singer actually meant their advice to be taken seriously and implemented. (3) The justification, based on emotional and financial burdens and suffering, is sincere, albeit commodifying in the sense criticized by Swift. (4) What lies underneath Kuhse and Singer’s thesis cannot be captured in one dimension. The utilitarian motives of ending useless suffering and easing burdens are present, but there are others: a reluctance to see a moral difference between killing and letting die; an aversion to giving humanity pride of place; and an irritation toward allocating resources to the few in more affluent countries at the expense of the many in less affluent ones.

## A recent controversy: killing unwanted infants

Alberto Giubilini and Francesca Minerva published in 2013 an article titled “After-birth abortion: why should the baby live?” [8] In the article, they described an anomaly that they perceived in reproductive policies. Pregnancies may be terminated legally at late stages, apparently because fetuses do not have the same intrinsic value that adult human beings, as persons, have [9, 10]. Yet infanticide is outlawed, although very young infants are not persons, either [11]. This generates, Giubilini and Minerva argued, a problem. A woman can find herself in drastically altered circumstances after she has given birth to an originally wanted child, but the only choices available to her are to keep the child or give it to adoption. Both alternatives can be psychologically too taxing on her. If they are, Giubilini and Minerva suggested, the woman should have the option of “after-birth abortion”—to have the infant killed by medical professionals.

The seriousness of this suggestion—whether the authors meant it or not—is a matter of some dispute. Minerva has, later on, stated that the idea was purely hypothetical, or conditional [12]. If abortion is allowed on psychological, social, and economic grounds, if newborn infants have the same moral status as fetuses, and if no other factors (especially the possibility of adoption after birth) create a moral distinction between abortion and infanticide, infanticide should be allowed on psychological, social, and economic grounds. In this case, no scandalous claim has been made, and there would be nothing to see here. The hypothetical formulation, which is also tautological, has been around for a long time [9–11]. Restating it would have added nothing new to bioethical discussion.

The article’s wording supports, however, a different interpretation [13], namely that the claim was assertive in the Kantian sense of assertive imperatives [14]. Here the first two of the “ifs” in Minerva’s formulation are considered true and the only conditional “if” which is often satisfied, is the last. This changes the message considerably. If abortion is allowed on psychological, social, and economic grounds, if newborn infants have the same moral status as fetuses, and *since* no other factors (especially the possibility of adoption after birth) create a moral distinction between abortion and infanticide, infanticide should be allowed on psychological, social, and economic grounds. I will take this to be Giubilini and Minerva’s argument, as did their defenders and critics in the ensuing debate [12, 13, 15].

The elements of the case are as follows. (1) A description of what Giubilini and Minerva see as a realistic situation: A woman, after giving birth, neither wants to keep her baby nor give it up to adoption, but, instead, wants the child to be killed. The law does not grant her wish and she is left in distress. (2) A genuine proposal to improve the situation by allowing her to have the infant killed. (3) A sincere *ad hominem* justification of the suggestion based on consistency in the treatment of unborn and born nonpersons. The *ad hominem* refers to the first two remaining “ifs” in Minerva’s argument. The conclusion does not follow if you do not condone abortion and believe that the unborn and the born are axiologically equal. (4) Underneath, Giubilini and Minerva’s desire to reduce suffering and their indignation over the logical inconsistency of denying the “after birth abortion” choice although there is no moral difference between taking the lives of late fetuses and very young infants.

## Another modern classic: making the best babies we can

Julian Savulescu published in 2001 a defense of eugenics that soon became seminal, “Procreative beneficence: why we should select the best children” [16]. He started by observing that people are genetically disposed to many diseases, conditions, and behaviors that have an impact on their lives. He went on to note that quite a few of these dispositions can be detected in prenatal tests on embryos or fetuses. He argued that there is often a connection between the predisposition, medical or nonmedical, and the quality of the individual’s life, if born.

Savulescu then proposed that “couples (or single reproducers) should select the child, of the possible children they could have, who is expected to have the best life, or at least as good a life as the others, based on the relevant, available information” [16, p. 415]. This, according to him, could be achieved by testing embryos and fetuses to gain all the genetic information available, including information about non-disease genes, and using it as the basis of decisions. Potential parents should not knowingly select an embryo or fetus with a detected future disease or disability or proneness to undesirable behavior. Savulescu provided a list of traits and behaviors that can be genetically detected: aggression and criminal behavior, alcoholism, anxiety and anxiety disorders, attention deficit hyperactivity disorder, antisocial personality disorder, homosexuality, maternal behavior, memory and intelligence, neuroticism, novelty seeking, schizophrenia, and substance addiction [16, p. 417]. Since some of the items do not sound like something to be avoided [17], decisions concerning their desirability would have to be made. Parents should not, however, choose an embryo or a fetus which would grow up to be a person with bad qualities if the alternative is to choose an embryo or a fetus which would grow up to be a person without these qualities.

Savulescu then offered several detailed justifications that all amount to the same thing. Potential parents should make decisions that maximize reasonably expected good. Reasonable expectations are best anchored in genetic information because it is the most reliable way of acquiring information about the quality of future lives. The good to be maximized is the quality of future lives in terms of lack of avoidable disease, disability, or unwanted behavioral tendencies.

The elements of Savulescu’s contribution are the following. (1) A description of the human condition from the viewpoint of biology: many things that can make our lives better or worse are, at least partly, predetermined by our genetic constitution. Up until now, we have not been able to avoid bad determinants, and this has caused much suffering and discomfort. (2) A genuine proposal to select children prenatally in the light of genetic testing. (3) A factual justification stating that genetic tests can reliably predict the probability of a good life; and a normative justification arguing that parents have a moral obligation to choose the children with the best lives they can. (4) Underneath, a eugenic belief in a better race and a utilitarian commitment to maximizing reasonably expected good.

## An innocuous suggestion: antinatal counseling for all

In 2004, I published myself a short note titled “A rational cure for pre-reproductive stress syndrome” [18]. The proposer, to be distinguished from me, opened the proceedings by stating:

Many women and men, usually in the 20–50 age cohort, suffer from a condition that can be called prereproductive stress syndrome. The primary symptom is that these individuals have an urge to have children. Secondary symptoms, which are many and varied, can include a conviction that this urge is self-evidently reasonable, and an illusion that others should help them in satisfying it. The syndrome typically disappears, at least for the time being, when the urge is met, and the standard treatment is, accordingly, pronatal counselling [18, p. 377].

I then went on to challenge the rationality and morality of having children in the first place. The conclusion was:

Possible parents could be told that, according to at least one philosopher, it would be all right for them not to reproduce at all. In a social environment where the pressure to procreate makes the choice in the majority of cases less than fully autonomous, this could empower people to make the unpopular, but if my arguments are sound, rational choice, to remain childless. In effect, this would cure their prereproductive stress syndrome [18, 378].

The conclusion concerns only rationality, not morality, and it is not entirely clear who drew it—the proposer, I, or both. Let me explain this last point.

I took in the article rationality to mean “maximin” caution in the spirit of John Rawls and his theory of justice [19]. We should always avoid the action alternative that in the worst-case scenario would produce the worst possible result. We should, in other words, minimize the probability of maximum harm. The worst possible result of human reproduction is, I maintained, to produce a child whose life turns out to be miserable. Since making babies always carries this risk of maximum harm, I further maintained, it is always irrational.

I took morality to mean that we should not bring about or allow avoidable suffering. Since all human beings suffer at one time or another during their lives, parents violate this rule, and their decision to have children is immoral. These views on rationality and morality were mine. I refrained, however, from preaching the immorality of reproduction to parents because that would probably cause suffering without reaching the desired aim, and therefore go against my own ethic [20; cf. 21]. I was left, then, with irrationality, which I thought could be safely conveyed to people who plan to have children under peer or other social pressure. The form of the message is a separate question. Did I really claim that “at least one philosopher” having an opinion on childbearing would be a persuasive argument? I doubt it. So, the formulation, like the introductory description of the “syndrome,” must be credited to the narrator.

To align my thesis with the other four, its elements are as follows. (1) A description of a mock syndrome for people’s wish to have children and a cure for it in pronatal counseling. (2) A genuine, if non-seriously worded, suggestion for turning the tables by non-directive antinatal counseling. (3) A sincere defense of the suggestion in terms of maximin rationality and a ban on causing avoidable suffering. (4) Underneath, my antinatalism and negative utilitarianism [22, p. 186].

## A comparison of the theses: grievances, proposals, and justifications

Let me pause here for a moment to take stock of the similarities and differences in the five theses, A-E. They are depicted in Table 1.

1. The seriousness of the grievances raised in the theses form a descending scale from Swift through Kuhse and Singer, Giubilini and Minerva, and Savulescu to me. The plight of the poor in Ireland was real, tangible, and unignorable. The suffering of some severely disabled infants, and the burden on their parents and society, are something to be reckoned with, in one way or another. The agony of mothers in altered circumstances can be real, but it is presumably rare and, according to popular sensibilities, somehow wrong, or strange. Genetically imperfect babies are born, but only eugenic scientists and philosophers seem to think that there is something amiss. Babies with or without disabilities or genetic perfection are born all the time, but only a handful of antinatalist academics and activists see it as a problem.
2. The proposals made by Swift, Kuhse and Singer, and Giubilini and Minerva are radical and involve killing babies. Swift stands apart on two accounts. His suggestion is by far the most revolting and scandalous of the three, but it is also ironic, not what the author actually means. Savulescu’s suggestion is divisive but not particularly radical. Societies already accept selection and terminations that aim to eliminate debilitating diseases, and the step from there to pre-empting other allegedly harmful conditions may be seen as one of degree. My suggestion to cautiously remind prospective parents that they are not under an obligation to have children might introduce a slightly unexpected form of counseling but does not even register on the gauge of repulsion that the first three do.
3. All these are justified by utilitarian considerations. What that means, however, comes in two different packages. According to a colloquial understanding, considerations are utilitarian if they involve—typically economic—calculations of what is useful and profitable. These are present in Swift’s mock justification, in Kuhse and Singer’s language of burdens, and perhaps indirectly in Savulescu’s eugenic ideas. According to a more theoretical understanding, utilitarian deliberations revolve around the greatest possible measurable, calculable, and comparable good. This good does not need to be economic or otherwise one-dimensional, but the requirements of measurability, calculability, and comparability tend to push analyses toward monism, with preference satisfaction, wellbeing, or health as a focal value. All five theses seek their legitimation from this model, with variations. In Swift’s time, utilitarianism as a philosophical doctrine was only just emerging [20], but he captured well its forthcoming hedonism and economic orientation. Kuhse and Singer, as well as Giubilini and Minerva, employed the time-honored utilitarian strategy of letting facts, as they perceive them, speak for themselves and concentrating on the inadequacy of the alternatives. Savulescu and I elaborate on the utilitarian core themes and distinguish between rationality, morality, and the law, but remain within the confines of the theory, despite my partial straying into the maximin territory.

**Table 1:** Similarities and differences in theses A-E

	(A) Swift	(B) Kuhse & Singer	(C) Giubilini & Minerva	(D) Savulescu	(E) Häyry
(1) Grievance	Serious	Not inconsiderable	Rare Strange	General Ideological	Non-existent
(2) Proposal	Scandalous Ironic	Debated Not ironic	Drastic Not ironic	Divisive Not ironic	Unexpected Not ironic
(3) Justification	Utilitarian Ironic	Utilitarian Not ironic	Utilitarian Not ironic	Utilitarian Not ironic	Utilitarian Not ironic
(4) Underneath	Sanctity Dignity	Anti-dogmatism	Anti-dogmatism	Positive Eugenics	Antinatalism Extinction
(5) Criticism	Dogmatic Tasteless	Reifying Ableist	Reifying Unnatural	Reifying Ableist	Absurd Nihilist

## A further comparison of the proposals: underneath and criticism

4. What lies underneath Swift’s proposal is diametrically opposed to what motivates Kuhse, Singer, Giubilini, and Minerva. Swift clearly believes in the sanctity, dignity, and intrinsic worth of human lives, and rejects economic calculations and social engineering that reify and commodify people. The other four condemn, as groundless dogmatism, all doctrines that assign exceptional value to humanity or traditional moral distinctions; and they assess practices based on the wellbeing and preference satisfaction they entail. The background of Savulescu’s view is in scientific enthusiasm and “positive” utilitarianism (maximizing wellbeing). These provide the foundation of his eugenics and quest for perfection. My suggestion has its roots in the theory of “negative” utilitarianism (minimizing suffering), with which I have flirted [20, pp. 122–124; 23]. Since suffering exists as long as human and other sentient beings exist, antinatalism and extinctionism are logical conclusions of the view. In critical literature, this outcome has been used as a *reductio ad absurdum* argument against it [24, p. 304 n. 62].
5. Utilitarians hold a specific view on dogmatism, a view that was formalized by Henry Sidgwick in his 1874 classic *The Methods of Ethics* [25]. Sidgwick argued that utilitarianism (as well as rational egoism) is supported by our self-evident moral intuitions, but that these should not be confused with our dogmatic intuitions. The former, according to Sidgwick, are justice (we should judge like cases alike), prudence (we should give all parts of our conscious lives equal value), and the universality of goodness (we should give everybody’s happiness the same weight) [25, pp. 381–2]. Universal hedonism (i.e. utilitarianism) is in line with all three and should be seen as the best moral theory overall (although rational egoism cannot be ruled out, either). A reliance on dogmatic intuitions like the sanctity of life is, however, mistaken. Dogmatic intuitions do not form a coherent whole and they may contradict one or several of the self-evident intuitions.

Sidgwick’s idea is prevalent among consequentialist ethicists, and it is employed to rule out not only commonsense intuitions but also entire moral systems. If Kantian maxims on human dignity or Aristotelian convictions on human flourishing do not fit the utilitarian mold, they, too, are dogmatic and unworthy of consideration. On this logic, at least Kuhse, Singer, Giubilini, and Minerva could criticize Swift for his dogmatism, as he discards a perfectly rational solution to a social problem. They could then turn the satire into a controversial view in their own sense by removing the irony and making the proposal genuine.

Apart from the charge of reification (turning people into things and objects of calculation), the consequentialist views have faced more specific criticisms. Kuhse and Singer’s plans for institutionalizing infanticide and Savulescu’s prompt to select children genetically can be seen as ableism—preferring people without known disabilities to people with them [26]. Giubilini and Minerva’s suggestion could perhaps be characterized as unnatural, but this needs specification. The word has many meanings and its relationship with immorality is contested [27]. Here the unnaturalness, as seen by Giubilini and Minerva’s critics, could mean deviating from the ideal of motherhood. Parents should, the criticism goes, love and accept their children as they are, and external factors should not change this [28].

My thesis is in a category of its own. All the others are pronatal. Irish Catholics should be allowed to have children even if they cannot afford to provide for them. Parents should be allowed to get rid of their severely disabled offspring so that they can have new children. Women in altered situations should be allowed to discard their unwanted babies to retain their willingness to reproduce. Parents should have perfect children, but if this is not possible, the best available children will do. Against all these, my thesis was that people should not have children at all. This is not dogmatic in any identifiable sense, nor is it ableist as it does not distinguish between better or worse brood. It could be called unnatural, if childbearing is the norm, but charges of absurdity and nihilism are even more tempting possibilities. If human life has no value, then what value can there be in anything?

## Senses and levels of controversiality

The five theses are controversial in different ways and on different levels.

1. The first point of contention can be the grievance. Swift’s description of the plight of the Irish poor was uncontestable, but his adjacent allegation that the English overlords were to blame could be challenged by claiming that something in the Irish Catholics themselves caused the situation. In Kuhse and Singer’s case, the fact that severely disabled babies are born and that some of them have little more than suffering in their lives is a fact, but open to interpretation. Maybe suffering gives human life its value or maybe the medical professionals’ reaction is the best, and there is no problem to be solved. Giubilini and Minerva’s concern is rare and its very existence is difficult to comprehend. Our popular imagery does not contain a portrait of a calmly and composedly infanticidal mother. Savulescu is correct in noting that people with predictable difficulties are allowed to be born, but what do we know about the goodness of these lives and is not diversity a value, anyway? In my thesis I am, of course, correct in observing that people reproduce, but almost no one sees this as an issue.
2. Swift’s proposal is gruesome, but he does not mean it, so the only criticism there could be that it is in bad taste to air such ideas. This also applies to the other views. Anyone who suggests, on whatever grounds, that babies should be deliberately killed, or let die, or denied birth, steps outside what is seen as decent conversation. This gives rise to indignation which goes further than the—also possible—theoretical condemnation of utilitarian moral theories.
3. Since Swift does not stand by his utilitarian justification, it is difficult to see cause for alarm here. All the others are serious in their reasoning, so moralists from competing schools can questions their views, but this is a sign of diversity in ethics and political theory, not controversy in any specific form. Although utilitarians disagree with Kantians and Kantians with Aristotelians and so on, we do not usually investigate these collisions as particularly controversial incidents.
4. The ideological undercurrents of the theses reflect the viewpoint conflicts already seen in the description of grievances. Some believe in the sanctity of life and others in utility calculations, and the opposition’s ideas are rejected. The clash may be more than a theoretical disagreement, though, and a source of genuine controversy. If people on one side of the debate are convinced that people on the other side are not only wrong but somehow wicked, the polemics between them may escalate beyond pure academic argumentation.
5. Accordingly, the criticisms levelled at opposing proposals can assume an air of hostile righteousness, and this can become the core of the controversy. The polemic then transcends polite doctrinal disagreement. Differences could be discussed as theoretical disputes, but indignation and a sense of evil in the rival views may take over. Views are seen as controversial not only because they deviate from “ours” but also because they are intellectually (traditional dogmas) or morally (challenges of traditional dogmas) bankrupt.

## Some lessons to be learned from Juvenalian and other satires, maybe

All through my analysis, Swift’s mock proposal has offered a point of comparison to contemporary controversial views. It is a Juvenalian satire, which means that it is a savage endeavor to ridicule public practices and make them objects of contempt. The message is that these practices are not only incompetent and mistaken but monstrous and evil. The mock proposal shows what the culprits are capable of and “hides” the moral condemnation of the author. This is in stark contrast with two other kinds of satire. Horatian ones represent a type of comedy. They describe human follies in a humorous and gently knocking manner, without accusing tones or anger. Menippean ones approach, in a distancing and playful way, social philosophy and social science. They criticize stagnated mental attitudes by drawing caricatures of authority figures and reminding that they, too, are mortal, and their views fallible [2–4].

The views expressed in theses B-D would probably fare better if they were flipped upside down and turned into Juvenalian satires. By mockingly proposing that babies can be exterminated at will and eugenic societies created [29] by parental guidance they would remind readers of the fundamental morbidity of utilitarian thinking. As they stand, their lack of distancing tends to infuriate people with traditional ideas. This is not ideal if they were meant to be starting points for reasoned discussion—an outcome which at least Singer and Minerva seem to hope [12, 30].

As a way forward, I submit that it could be good for bioethicists with controversial views to show some sense of humor and modesty. I arrive to this conclusion by considering my own contribution to the debate and its reception [18]. Judging by the two excerpts that I cite above, it seems that I hit, quite inadvertently, the spirit of Horatian satire, first in the description of the case as a parody of a medical syndrome and then in the proposal that parents be told about one philosopher’s views as a part of nondirectional counseling. As a result, all responses to the article, including critical ones, have been amenable. My preposterous and sincere suggestion to let humankind fade away has earned no threats, on the contrary, the idea, or some more palatable form of it, has drawn a modicum of polite interest. If that is the point of presenting controversial views, a less self-serious approach might be worth trying, maybe with an awareness of the ironic and satirical potential of any proposal that can shock wider audiences.
